# Desflurane Allows for a Faster Emergence When Compared to Sevoflurane without Affecting the Baseline Cognitive Recovery Time

**DOI:** 10.3389/fmed.2015.00075

**Published:** 2015-10-28

**Authors:** Joseph G. Werner, Karina Castellon-Larios, Cattleya Thongrong, Bodo E. Knudsen, Deborah S. Lowery, Maria A. Antor, Sergio Daniel Bergese

**Affiliations:** ^1^Department of Anesthesiology, The Ohio State University Wexner Medical Center, Columbus, OH, USA; ^2^Department of Urology, The Ohio State University Wexner Medical Center, Columbus, OH, USA

**Keywords:** sevoflurane, desflurane, emergence, outpatients, cognition

## Abstract

**Aims:**

We compared the effect of desflurane and sevoflurane on anesthesia recovery time in patients undergoing urological cystoscopic surgery. The Short Orientation-Memory-Concentration Test (SOMCT) measured and compared cognitive impairment between groups and coughing was assessed throughout the anesthetic.

**Methods and materials:**

This investigation included 75 ambulatory patients. Patients were randomized to receive either desflurane or sevoflurane. Inhalational anesthetics were discontinued after removal of the cystoscope and once repositioning of the patient was final. Coughing assessment and awakening time from anesthesia were assessed by a blinded observer.

**Statistical analysis used:**

Statistical analysis was performed by using *t*-test for parametric variables and Mann–Whitney *U* test for non-parametric variables.

**Results:**

The primary endpoint, mean time to eye-opening, was 5.0 ± 2.5 min for desflurane and 7.9 ± 4.1 min for sevoflurane (*p* < 0.001). There were no significant differences in time to SOMCT recovery (*p* = 0.109), overall time spent in the post-anesthesia care unit (PACU) (*p* = 0.924) or time to discharge (*p* = 0.363). Median time until readiness for discharge was 9 min in the desflurane group, while the sevoflurane group had a median time of 20 min (*p* = 0.020). The overall incidence of coughing during the perioperative period was significantly higher in the desflurane (*p* = 0.030).

**Conclusion:**

We re-confirmed that patients receiving desflurane had a faster emergence and met the criteria to be discharged from the PACU earlier. No difference was found in time to return to baseline cognition between desflurane and sevoflurane.

## Introduction

Desflurane and sevoflurane are the two most commonly administered inhaled anesthetics for outpatient surgeries due to their favorable pharmacokinetic profiles and low incidence of untoward effects. Both of these agents have been safely used for anesthesia maintenance using a laryngeal mask airway (LMA) (1–4). Multiple studies have demonstrated that desflurane allows for a more rapid emergence than sevoflurane, and this may be beneficial for outpatient surgery, where quick case turnover and reduced post-anesthesia care unit (PACU) time is essential to ensure a good workflow (2, 3).

Our primary objective was to compare the effect of desflurane and sevoflurane on anesthesia recovery time in patients undergoing urological cystoscopic surgery under general anesthesia using a LMA. Given the recent interest in post-operative cognitive dysfunction, as a secondary objective, we employed a more robust test of cognitive function to see if there was a difference in recovery of cognitive function between the two inhalational anesthetic, using the Short Orientation-Memory-Concentration Test (SOMCT) (Supplementary Material) (5, 6). Additionally, we measured the presence of coughing, during the period from induction to return of spontaneous respiration. In order to accomplish this, we modified our anesthesia protocol to maintain stable end-tidal anesthetic concentrations and administer intravenous (IV) fentanyl on induction.

## Materials and Methods

We conducted a single center, prospective, randomized clinical trial after obtaining Institutional Review Board approval and being registered in clinicaltrials.gov under the identifier NCT01219881.

The investigation was conducted between September 2010 and October 2012 on 75 ambulatory patients ages 50–75, ASA I to III, and undergoing urological cystoscopic surgery under general anesthesia with a LMA. After patients signed consent, they were randomized via a computer-generated list to receive either desflurane or sevoflurane for induction and maintenance of anesthesia. Principal investigator and co-investigators were able to enroll patients into the study. The exclusion criteria included history of allergy, intolerance, or contraindications to any study medication, alcohol or drug abuse within 1 year, body mass index >35kg/m^2^, history of hiatal hernia, gastroesophageal reflux disease, or pharyngeal pathology, and a diagnosis of decreased pulmonary compliance.

Demographics, detailed medical history, current medications, and baseline vitals were collected from each patient. Prior to entering the operating room, each patient was asked to complete a baseline 11-point verbal rating questionnaire for pain and nausea.

After premedication with 0.5–2 mg IV midazolam, patients were preoxygenated using spontaneous mask ventilation at 100% O_2_ and a fresh gas flow of 6 L/min of oxygen for 1–4 min prior to induction. Anesthesia induction was performed via IV bolus injections of 2 mg/kg propofol and 1–2 μg/kg fentanyl. After induction, the inhaled anesthetic vaporizer was opened to 1 minimum alveolar concentration (MAC), i.e., 5.4–7.4 volume% for desflurane and 1.4–2.5 volume% for sevoflurane, based on study randomization for 1–2 min using mask ventilation with inhaled anesthetic; following, the fresh gas inflow was turned off, and a lubricated LMA was inserted. After placing a seal around it, and measuring for tidal volume leak, the fresh gas inflow was immediately restored to 4–6 L/min of 100% oxygen with the vaporizer set at 1 MAC for 3 min. The patient was placed on volume or pressure-control mechanical ventilation until spontaneous respiratory efforts were made and spontaneous breathing without support was achieved. For the duration of the case, desflurane or sevoflurane was maintained between 0.5 and 1 MAC, inspired in a 50:50 air/oxygen mixture at a total gas flow rate of 1 L/min.

In addition to American Society of Anesthesiologists-recommended standard monitoring, we used continuous registration of bispectral index (BIS) in all patients. The hemodynamic, oxygen saturation and BIS values were recorded before induction of anesthesia and every 5 min throughout surgery. IV fluids, additional boluses of fentanyl, lidocaine, propofol, or vasopressors, were administered as needed to maintain analgesia and hemodynamics within 20% of baseline. Opioid medication was also used according to anesthesiologist discretion. IV hydromorphone or morphine was used. These were then transformed to morphine equivalents later on for the purpose of this study.

Each episode of coughing from induction of anesthesia through removal of LMA was recorded by a blinded observer and graded according to a standardized scale (2). Coughing was characterized as 0 = no coughing; 1 = single cough and SpO_2_ ≥ 95%; 2 = multiple coughs occurred and SpO_2_ ≥ 95%; 3 = multiple coughs occurred and SpO_2_ < 95%; 4 = multiple coughs occurred, SpO_2_ < 95%, and if coughing required administration of an IV medication.

At the end of procedure, the inhalational anesthetic was discontinued and the fresh gas flow was increased to 6–8 L/min of 100% oxygen. Discontinuation only occurred after removal of the cystoscope and once repositioning of the patient was final. In order to standardize the awakening, the only permissible stimulus was verbalization of the patient’s name and the command “open your eyes,” which was given at 1-min intervals while the LMA was still in place. The mask was removed once the patient displayed a purposeful response to the verbal stimuli. Coughing assessment and awakening time from anesthesia were assessed by a blinded, trained observer.

Post-anesthesia physical recovery status was evaluated according to the Aldrete score, assessed every 5 min after extubation until the patients reached a score ≥9 (7). Thereafter, a blinded observer performed the SOMCT every 15 min to evaluate post-operative cognitive function for the first hour after surgery and at the time of discharge from the PACU (8). Procedure length, length of stay in the PACU, and time to discharge from the hospital were also recorded.

At 24 h after surgery, overall satisfaction about their experience with the anesthetic medication was assessed; either by personal interview or phone call conducted by blinded personnel. We assessed their subjective well-being while in the PACU, as well as their pain treatment and any intraoperative awareness. Satisfaction was rated using a 3-point rating scale: 2 = highly satisfied, 1 = satisfied, and 0 = dissatisfied.

The sample size of 68 patients was need to reach an 80% power and to detect an intergroup difference of 3 min in the time to eye-opening after surgery between the two treatment groups assuming group means are 5.0 and 8.0 with a SD of 3.0 and 5.0, respectively. The sample size was calculated based on a two-sided, two-sample *T* test at the significance level of 0.05.

Computer software SAS 9.2 was used to conduct the statistical analysis. Initially, descriptive statistics were calculated for demographic data and presented as mean ± SD, median, or percentages. Statistical analysis was performed by using *t*-test for parametric variables and Mann–Whitney *U* test for non-parametric variables. Additionally, chi-square and Fisher’s exact tests were used to analyze count data to investigate the relationship between categorical variables. Log transformation has been performed whenever found to be appropriate (PACU length of stay and length of surgery) before the analysis. A *p* < 0.05 was considered statistically significant.

## Results

Seventy-five patients provided written consent for the study, 66 (*n* = 34 desflurane group; *n* = 32 sevoflurane group) were able to successfully complete all the assessments. The primary reasons for screen failure were failure to meet all the inclusion/exclusion criteria (*n* = 3) and failure to correctly place the LMA (*n* = 4). There were two cases that were considered early termination and not included in the final analysis: one patient was converted to the prone position (intubated midway through the case); the other patient experienced a laryngospasm prior to case completion.

There were no significant differences in demographic parameters except pain at rest (Table 1). There were four subjects with pain at rest in the desflurane group (12%) versus 11 in the sevoflurane group (34%) (*p* = 0.028). No significant differences in anesthetic medications administered were observed between the two groups (Table 2).

**Table 1:** **Demographic data**.

	Desflurane (*n* **=** 34)	Sevoflurane (*n* **=** 32)	*p*-Value
Age (years old)	61.1 ± 7.7	61.3 ± 6.6	0.899
Weight (kg)	83.3 ± 18.2	81.3 ± 16.6	0.646
Height (cm)	172.2 ± 10.7	170.4 ± 9.5	0.484
BMI (kg/m^2^)	27.8 ± 4.6	27.8 ± 4.5	0.952
Sex (male) (*n*, %)	20 (58%)	15 (46%)	0.331
ASA (*n*)	I: 1	I: 0	0.070
	II: 13	II: 16	
	III: 20	III: 16	
History of smoking (yes) (*n*, %)	16 (47%)	10 (31%)	0.189
Baseline verbal rating scale scores (0–10) (*n*, %)			
Nausea (yes)	2 (6%)	3 (9%)	0.592
Pain at rest (yes)	4 (12%)	11 (34%)	**0.028**^a^
			
Baseline SOMCT score (maximum 28) [median (IQR)]	24 (24, 26)	24 (24, 26)	0.939
Anesthesia time (min)	82.7 ± 15.0	85.9 ± 21.9	0.491
Surgery time (min)	45.0 ± 16.9	45.7 ± 17.0	0.829

*^a^Indicates significant differences between two groups*.

*Data are presented as mean ± SD or number (percentage) unless otherwise noted*.

*Bold font means statistically significant measurements between both groups*.

**Table 2:** **Anesthetic medications**.

Medication dosage	Desflurane	Sevoflurane	*p*-Value
Midazolam (mg)	2 (2, 2)	2 (2, 2)	0.821
Fentanyl (μg)	100 (100, 100)	100 (75, 100)	0.628
Propofol (mg)	165 (140, 200)	150 (132, 179)	0.438
Lidocaine (mg)	100 (80, 100)	90 (60, 100)	0.280
Total opioid consumption (mg)	37 (30, 52)	42 (37, 65)	0.062

*No significant differences were observed*.

*Data are presented as median [interquartile range (IQR)]*.

Results from the recovery endpoints are presented in Table 3. The mean time to eye-opening was 5.0 ± 2.5 min for desflurane and 7.9 ± 4.1 min for sevoflurane (*p* < 0.001). There were no significant differences in time to SOMCT recovery (*p* = 0.109), overall time spent in the PACU (*p* = 0.924), or time to discharge (*p* = 0.363). Median time until readiness for discharge was 9 min [mean of 5–18 min in the desflurane group, median of 20 min with mean of 9–27 min for the sevoflurane group (*p* = 0.02)].

**Table 3:** **Emergence and immediate recovery times after discontinuation of volatile anesthetics in the two study groups**.

Duration^a^	Desflurane	Sevoflurane	*p*-Value
Time to eye-opening (extubation time)	5.0 ± 2.5	7.9 ± 4.1	<0.001*
Time until Aldrete ≥9	5 (0, 5)	5 (5, 5)	0.404
Time until SOMCT recovery (extubation time + return to SOMCT baseline)	15 (5, 30)	15 (5, 20)	0.109
Time until ready for discharge (extubation time + time until Aldrete ≥9)	9 (5, 18)	20 (9, 27)	0.020*
PACU length of stay	71.4 ± 31.6	71.7 ± 29.3	0.924
Floor length of stay	89 (75, 122)	115 (78, 130)	0.199
Time until discharge	141 (113, 189)	158 (130, 210)	0.363

*PACU, post-anesthesia care unit; SOMCT, Short Orientation-Memory-Concentration Test*.

*Values are number (*n*), percentage (%), mean ± SD or median (range)*.

*p < 0.05 was considered statistically significant.

*^a^Duration in minutes*.

*Bold font means statistically significant measurements between both groups*.

The incidences of coughing during induction of anesthesia, intraoperatively, or at emergence from anesthesia were individually not significantly higher in patients receiving desflurane; however, the overall incidence of coughing during the perioperative period was significantly higher in the desflurane group compared to the sevoflurane group (*p* = 0.030; Table 4). The coughing episodes that occurred were short-lasting, did not lead to desaturation below 95%, or interrupt the surgical procedures. As mentioned earlier, one case of intraoperative laryngospasm occurred in the desflurane treatment arm. No significant difference was observed with regard to the anesthesia satisfaction endpoint (Table 5).

**Table 4:** **The incidence of coughing during the perioperative period**.

Coughing	Desflurane	Sevoflurane	*p*-Value
Intubation (*n*, %)	4 (12%)	1 (3)	0.197
Degree (*n*, %)			
0	30 (88%)	31 (97%)	0.356
1	2 (6%)	1 (3%)	0.999
2	2 (6%)	0	0.492
Extubation (*n*, %)	10 (30%)	4 (13%)	0.060
Degree (*n*, %)			
0	23 (70%)	27 (87%)	0.132
1	4 (12%)	0	0.113
2	6 (18%)	4 (13%)	0.733
Coughed during intubation and extubation (*n*, %)	2 (6%)	1 (3%)	0.392
Number of patients coughing perioperatively (*n*, %)	12 (35%)	4 (12%)	0.030*

*Data are presented as number (percentage)*.

*p < 0.05 was considered statistically significant.

*Bold font means statistically significant measurements between both groups*.

**Table 5:** **Patient satisfaction rate**.

Category	Sevoflurane (*n* **=** 24)	Desflurane (*n* **=** 25)	*p*-Value
Very satisfied	11 (45.8%)	11 (44.0%)	0.8839
Satisfied	12 (50.0%)	14 (56.0%)	
Unsatisfied	1 (4.2%)	0 (0.0%)	

*Data were available for 24 and 25 patients in the sevoflurane and desflurane group, respectively*.

*Data are presented as number (percentage)*.

## Discussion

Given the blood gas-partition coefficients for desflurane and sevoflurane, it is not surprising that patients that received desflurane had quicker emergence from anesthesia, and the amount of time, roughly 3 min, is similar to that reported previously (4,  9). Furthermore, the time until the patients were ready for PACU discharge was also shorter by a median value of 11 min if the patient was administered desflurane. In a busy outpatient surgery center, the cumulative gain in time over 6–10 cases might encourage one to use desflurane for maintenance anesthesia for all the patients. However, the overall duration of PACU time did not differ between groups and our mean PACU length of stay of 71.4 and 71.7 min was similar to the length of stay to the study by White et al., which had mean times of 80 and 79 min for desflurane and sevoflurane, respectively. It is unlikely that this PACU length of stay could be shortened, especially in ambulatory surgery as it takes time for patients to display that they are “street ready” by being able to drink fluids without getting nauseated, tolerable pain levels, be able to ambulate, as well as have an Aldrete score ≥9. The primary outcome of our study is consistent with previous studies that demonstrate a quicker emergence with desflurane, with no effect on PACU discharge (3, 4, 10).

The SOMCT is a six-item test that can be used to stratify cognitive deficits. It is a shortened test that correlates with the scores of a larger validated 26-item test (Blessed Information-Memory-Concentration Test) and thus has the benefit of being able to stratify cognitive dysfunction yet short enough to be given multiple times to assess the duration of this cognitive impairment (8, 11). Although the SOMCT is validated in the elderly to help stratify dementia, it is also possible that it is not robust enough to detect early post-anesthetic cognitive impairment.

Bilotta et al. (6) showed quicker cognitive recovery after desflurane, in comparison with sevoflurane, as assessed with SOMCT in 56 patients undergoing craniotomy. In their study, the mean body mass index values were 27.7 and 28.1 for desflurane and sevoflurane, whereas our study had essentially the same mean body mass index of 27.8 for both groups. We were unable to demonstrate any difference in recovery time to baseline SOMCT scores. Therefore, it is unlikely that the body habitus (and inhalational anesthetic retention) is the cause for the differences in cognitive impairment between the two studies. It is possible that the anesthetic duration that was nearly twice longer in their study and, presumably, the specific characteristics of the patient group undergoing craniotomy for resection of supratentorial lesions may have influenced the slower cognitive recovery in the sevoflurane group.

Other studies have utilized the mini-mental status exams (MMSE) and digit-symbol substitution tests to assess cognitive impairment, with multiple studies showing no difference in cognitive impairment using these tests (2, 10, 12–14). Furthermore, the MMSE has been shown to have a sensitivity of 87%, a specificity of 82%, and a false positive ratio of 39% when used to detect dementia and delirium on a general medicine floor (15). Based on the positive findings from Bilotta et al., we decided that the SOMCT would be a more practical test for assessing post-operative cognitive impairment on a timely basis. As with any test that is repeated over time, one is concerned with test–retest reliability and the practice effect, which could mask cognitive impairment (16). Our patient population included both middle-aged and elderly patients with a mean age of 61 years, so it is possible that observation of impairment was reduced secondarily to the inclusion of relatively younger patients. Future studies are needed to validate the SOMCT for post-operative assessment of cognitive dysfunction in elderly patients (over 65 years) and compare its usefulness with other relevant neuropsychological tests.

Multiple researchers have studied airway irritation as a secondary outcome when comparing desflurane with sevoflurane. In a recently published study, the amount of premedication and opiates administered to patients had been limited with an attempt to expedite patient discharge and diminish post-operative nausea and vomiting (4). The authors investigated coughing at induction, during the surgery, and extubation and reported that although coughing at each phase was not different between desflurane and sevoflurane groups, the combined cumulative incidence was significantly higher in the desflurane group. Our study confirmed the validity of their results showing a similar, statistically significant increase in the incidence of cumulative perioperative coughing in the desflurane group. It has been shown that administering opioid on induction can diminish the airway irritability associated with desflurane (16). All patients in our study received 1–2 μg/kg of fentanyl on induction, and although it diminished the initial airway irritation associated with desflurane, it did not reduce overall coughing observed in the perioperative period. Of note, patients in the sevoflurane group had a higher preoperative incidence of pain and the amount of opiates administered to this group was marginally significantly increased (*p* = 0.062), which may have influenced the cumulative incidence of coughing. Saros et al. probably incorporated the most efforts to diminish airway irritation with desflurane (3). Despite these, stridor was noted in 5/35 patients receiving desflurane versus 1/35 patients that received sevoflurane for anesthesia maintenance using a LMA. In our study, one patient that was anesthetized with desflurane had a laryngospasm and required management that deviated from our study protocol and thus was excluded from data analysis. A randomized prospective study with airway irritation as the primary outcome should be performed to more definitively address this question.

Our study design also utilized 1 MAC of inhalational anesthetic to ventilate patients prior to LMA insertion and kept the machine primed with gas by turning off fresh gas inflow and leaving the vaporizers turned on during the interval between masking, LMA insertion, and reattachment of the breathing circuit. Furthermore, we placed the patient on mechanical ventilation until they began spontaneous ventilation. By performing these steps, we ensured a consistent end-tidal anesthetic concentration throughout the induction period and prevented any potential sudden exposure of highly concentrated desflurane during the recovering period from induction to return of spontaneous ventilation. Due to the application of the aforementioned approach and use of fentanyl on induction, no difference was revealed between the two groups in incidence of coughing at induction. Although coughing at extubation itself was not significantly different between the two treatment arms, this is the phase in which most of the coughing events were observed. Perhaps a future study could look at administering fentanyl again 15 min prior to extubation to help attenuate the irritability around extubation.

## Conclusion

Our study confirmed that patients receiving desflurane had a faster emergence and met the criteria to be discharged from the PACU earlier; however, the total time spent in PACU was not different. No difference was found in time to return to baseline cognition between both groups; however, our study was not designed to address this question as a primary outcome. Lastly, desflurane caused more airway irritation than sevoflurane, despite the administration of fentanyl on induction and ensuring a consistent end-tidal anesthetic gas concentration during the induction period.

## Author Contributions

Guarantor of integrity of the entire study: JW. Study concepts and design: JW, SB, and MA. Literature research: KC-L and MA. Data analysis: KC-L and JW. Manuscript preparation: JW, KC-L, CT, BK, DL, and SB. Manuscript editing: KC-L.

## Conflict of Interest Statement

The authors declare that the research was conducted in the absence of any commercial or financial relationships that could be construed as a potential conflict of interest.
