# Supplementary material for: Desflurane Allows for a Faster Emergence When Compared to Sevoflurane without Affecting the Baseline Cognitive Recovery Time
Source: Front Med (Lausanne). 2015 Oct 28;2:75. doi: 10.3389/fmed.2015.00075 (PMC4623413; doi:10.3389/fmed.2015.00075)
Supplement: Supplementary file 1 [file table_1.pdf]

Appendix 1.

Short Orientation Memory Concentration Test*

Items	Question	Maximum Error	Score	Points	Total
1	What is the current year?	1		x 4	
2<br>*Memory Phrase*	What month is it now?<br>*Repeat this phrase after me:*<br>*John Brown, 42 Market Street, Chicago*	1		x 3	
3	About what time is it? (within 1 hour)	1		x3	
4	Count backwards from 20 to 1	2		x2	
5	Say the months of the year in reverse order	2		x2	
6	Repeat the memory phrase. Each of the following is worth 1 point:<br>-John<br>-Brown<br>-42<br>-Market Street<br>-Chicago	5		x2	

*Score ranges from 0 to 28. The higher the score indicates better cognitive function. Scores over 20 points are considered ‘normal’.
